# Corporate Social Responsibility Activities Through Twitter: From Topic Model Analysis to Indexes Measuring Communication Characteristics

**DOI:** 10.1007/s11205-022-02993-8

**Published:** 2022-08-20

**Authors:** Camilla Salvatore, Silvia Biffignandi, Annamaria Bianchi

**Affiliations:** 1 Department of Economics, Management and Statistics (DEMS), University of Milano-Bicocca, Piazza dell’Ateneo Nuovo, 1, 20126 Milan, Italy; 2 Department of Economics, University of Bergamo, Bergamo, Italy; 3 Bergamo, Italy

**Keywords:** Sustainable development, Corporate sustainability, Indicators, Structural topic model, Text mining, Social media

## Abstract

The communication of corporate social responsibility (CSR) highlights the behavior of the business toward CSR and their framework of sustainable development (SD), thus helping policymakers understand the role businesses play with respect to the 2030 Agenda. Despite its importance, this is still a relatively underexamined and emerging topic. In our paper, we focus on what businesses communicate about CSR through social media and how this relates to the Sustainable Development Goals (SDGs). We identified the topics discussed on Twitter, their evolution over time, and the differences across sectors. We applied the structural topic model (STM) algorithm, which allowed us to estimate the model, including document-level metadata (time and sector). This model proved to be a powerful tool for topic detection and the estimation of the effects of time and sector on the discussion proportion of the topics. Indeed, we found that the topics were well identified overall, and the model allowed catching signals from the data. We derived CSR communication indexes directly from the topic model (TM) results and propose the use of dissimilarity and homogeneity indexes to describe the communication mix and highlight differences and identify clusters.

## Introduction

Sustainable development (SD) was formally defined in the Brundtland Report as the “development that meets the needs of the present without compromising the ability of future generations to meet their own needs” (WCED, 1987). In the last few years, this concept has become increasingly important at the global level, especially after the Millennium Development Goals^1^ (2000) were established and the 2030 Agenda (United Nations, 2015) was launched, setting 17 Sustainable Development Goals (SDGs). For an overview of the evolution of the SD concept, approaches, and indicators, refer to Hák et al. (2016). Here, we focus our attention only on the concepts related to the 2030 Agenda. The SDGs put forth a global commitment to guarantee the protection of the planet and its inhabitants. The SDG framework consists of a collection of relevant goals, targets, and indicators, but it does not refer to a conceptual model. SDGs allow for describing the trends of each single indicator, but they pay attention neither to the trade-offs between dimensions nor to the effects that go beyond geographical and political boundaries. They involve actions from politicians, institutions, scholars, international agencies, businesses, and other stakeholders. To reach SDGs, in addition to the efforts of national and local governments, the efforts of civil society organizations and corporations are also indispensable. The role of multinational corporations, which currently cover two-thirds of global trade, is especially important (PwC, 2015).

Although the SDGs are not legally binding, they are the basic framework by which all United Nations’ (UN) member states can form future regulations and incentives. Incorporating SDGs into corporate strategies and goals will be crucial in the future. The UN is calling for active participation from companies to reach the SDGs. Activities such as donations and charitable events are no longer sufficient. Companies should help address the key issues highlighted by the SDGs by developing new and innovative ideas and business models. Now, including SDGs in the strategy has become an essential choice for companies to achieve sustainable success and competitive advantage. Since the 2030 Agenda is a plan of action, categories of information and initiatives emerging on social media (SM) seem particularly interesting and can help us understand how and to what extent people, businesses, and organizations are playing a more active role in contributing to SD.

SD is linked to the commitment of all the agents in society toward different aspects of progress, which clearly cannot be limited to the study and measurement of economic growth and performance based on the Gross Domestic Product (GDP) indicator. However, it should include different environmental and social dimensions (see Alaimo and Maggino (2020) and Alaimo et al. (2020), for an overview of the main theories). SD attains a complex research environment (Maggino, 2017), in a perspective of a multi-indicator system. As explained in Conegliaro’s (2021), different perspective can be considered with respect to SD and well-being.

The proliferation of SM due to technological advances has significantly changed communication among citizens, businesses, and institutions. These changes have increased public awareness of social development and environmental issues and have created greater transparency in corporate activities.

In order to formulate and implement concrete strategies for achieving SDGs, measuring and evaluating SDGs are essential for both governments and firms since they are useful decision-making tool in various areas of sustainability (Giambona & Vassallo, 2014). For this reason, several measurement studies exist in the literature, which are based on a variety of approaches either in terms of their methodology or with alternative theoretical perspectives, each reflecting on a specific point from the complex SDGs and SD in general. Listing the literature regarding the measurement of this general theme is beyond the scope of this paper. Let us just quote a recent paper based on websites and SM data. Lee and Kim (2021) proposed the novel SDG Social Index that will help reach SDGs, and it involves collecting the SM text data, instead of survey data, of one company. People’s comments on the company’s posts on Twitter, Facebook, and blogs are analyzed to understand how well it is doing in their aim of reaching SDGs. This could be considered for public reporting assessment.

The contribution of this paper is in the direction of studies about business communication and its role in corporate social responsibility (CSR) in the context of SD. Various perspectives, methods, and data sources can be used to study and measure CSR. In the following sections the perspective adopted in the paper and the general context are explained. The paper is organized in six sections. Section 2 describes the research context. Section 3 discusses the research aims and the data considered. Section 4 presents the methods implemented for the topic model (TM) and index construction. Section 5 discusses topic discovery, the effect of covariates, the link with SDGs, and the communication mix in firms and sectors. The main conclusions, along with considerations about future research, are presented at the end of the paper in Sect. 6. The Appendix section provides some more detailed results.

## Background and Research Context

In this paper, we focus on businesses with respect to SD, with particular regard to their impact on society. Businesses are economic agents that can and should play a role in the complex scenario of SD. For this reason, for example, the performance of businesses should not be measured only with respect to their economic and financial results. It is necessary to go beyond them. There are several aspects impacting society on the path toward a more sustainable world. Businesses are engaging in new initiatives and behaviors. Moreover, the idea of environmentally friendly products and cleaner production processes has become central in the business world. With respect to the social function of firms, they are transforming their role by focusing not only on philanthropic activities but also on initiatives that meet communities’ needs. SD and socially responsible behaviors are linked to the concept of CSR, which refers to the implementation of activities aiming to improve firms’ reputation and positively impact society.

This concept of CSR has a long history in the academic literature, which highlights its multi-dimensional nature. In this respect, the definition of CSR has been widely discussed. The dimensions of CSR and their measurement have been a main issue, and here are some examples of the dimensions. As Caroll (2015, 2016) recognizes, CSR emerged in the 1960s and, over the years, several variations and connected concepts have been developed, such as corporate social responsiveness and performance (1970–80 s), business ethics and stakeholder management (1980s); this also includes the more recent concepts of corporate citizenship, sustainability, conscious capitalism, and creating shared value (1990–2000s +). Several studies have proposed different definitions and numbers of CSR dimensions. For example, Caroll (1991) posited there were four dimensions: economic, legal, ethical, and philanthropic responsibilities. Dahlsrud (2008) analyzed CSR literature and found 37 different definitions that can be grouped into the following five dimension categories: environmental, social, economic, stakeholder-related, and voluntariness-oriented. Ingenhoff and Sommer (2011) proposed five categories: society, environment, employees, sponsoring, and voluntariness. More recently, Kim et al. (2014) studying CSR communication on a Social Media (Facebook) proposed six dimensions: environmental, philanthropic, educational commitments, community/employee involvement, public health commitments, and sponsorship of cultural/sport events. Alhaddi (2015) presents an overview of the main theories of the CSR and related dimensions, between them the triple bottom theory: economic, environment and social. Irrespective of the various above-mentioned specifications, the dimensions of CSR coherent with the SDG pillars and SD are in line with the triple bottom theory, i.e.: environmental, economic, and social. In our paper, we consider these three CSR dimensions, which fits the theoretical framework of the link between CSR and SDGs.

In addition to the discussion of the number of dimensions, the traditional literature is about the measurement—that is, the evaluation of the perspective considering the aggregated indicators of business strategies and actions. A direction to take in the literature on CSR is the study and development of rating scores, indexes, and rankings in order to measure the sustainability efforts and performance of firms (Diez-Cañamero et al., 2020; Dočekalová & Kocmanová, 2016; Vani, et al., 2016; Seibert, Macagnan, & & Dixon, 2021). Such indexes are mainly based on the construction of composite indicators to account for the multi-dimensional nature of CSR. The main sources are surveys and official statistics. Indexes differ in method, data source, and the theoretical perspective under study.

With regard to the perspective of sustainability communication, which is the theoretical framework of this paper, most studies were based on sustainability reports. Recent literature has been published after analyzing online communication and experimenting with websites or SM data to measure business communication about sustainability both at a general level and an independent business level. For example, Moreno and Capriotti (2009) deepen the understanding of corporate websites through content analysis—in which CSR, corporate citizenship (CC), and SD (also recognized as CSR/CC/SD) issues are included. At the time this study obtained its findings, the web, as an instrument of communication, offered limited content on CSR, which was not sufficiently coherent with the corporate behavior they reported.

The concept of CSR has evolved over time, and it is expected to evolve further according to societal changes and to continue to acquire new nomenclatures. In this paper, we do not delve into the historical analysis of CSR and the number of its dimensions. To simplify this task, we may distinguish between traditional and contemporary CSR. Traditional CSR refers to when a company generates its profits and creates value without much consideration for wider societal implications beyond shareholders and, at times, customers. According to contemporary CSR, businesses view responsible behavior as a means to generate profits while living up to society’s expectations. In other words, CSR is part of their daily business. It refers to when a corporation goes beyond making money and engages in actions that result in social good surpassing the interests of the corporation, which is required by law (McWilliams et al., 2006). Thus, in this paper, the contemporary view of CSR is assumed.

Social responsibility is an abstract attribute that cannot be directly observed in the products and/or services provided by organizations, which creates the need to disclose information about it to society. The informational efficiency of social responsibility requires the organization to establish strategies that focus on stakeholders. In other words, organizations should publish information that is of interest to key stakeholders, thereby establishing a competitive advantage and impacting society.

The availability of SM data provides an excellent opportunity to investigate online communication related to CSR activities and to construct innovative SM-based indicators. SM are both a Big Data (BD) source of information and a powerful communication tool for companies. Indeed, the diffusion of SM led to new opportunities, the first of which was the possibility of reaching a broader public base. Also, with respect to other mass and new media channels, communication on SM is different; it is amplified, and the public can actively participate in discussions. In particular, the communication of CSR activities, which plays a fundamental role in enhancing firms’ reputation, can enjoy the new opportunities derived from their use (Cho et al., 2017; Chae & Park, 2018; Lee, Oh, & Kim, 2013). While firms disclose their practices solely to stakeholders through CSR reports, they communicate, influence, and get feedback from a broader audience through SM. The definition of the target audience varies according to and within the selected communication channel. For example, CSR reports are technical and are addressed to a specific type of stakeholders, such as investors. Contrary to this, SM involves a broader audience. Companies can implement different communication strategies according to the target audience—for example, customers or younger people. Identifying key stakeholders to whom the communication must be addressed is essential in order to make it effective, gain a competitive advantage, and establish brand positioning. The study of SM for CSR purposes is a growing field in academic research (Araujo & Kollat, 2018; Saxton, Gomez, Ngoh, Lin, & Dietrich, 2019), and *listening* to online communication is useful to researchers and practitioners so they can obtain insights about the changes in business practices and monitor the behavior of the business in relation to the SD framework and 2030 Agenda. Despite its importance, this is still a relatively underexamined and emerging topic.

## Research Aims and Data

### Research Aims

The abovementioned issues show that businesses should and are contributing to collective SD efforts. To encompass this task, it is necessary for their strategies—especially their actions and communication—follow some guidelines and ethical rules, such as the Global Reporting Initiative (GRI), UN Global Compact, World Business Council for Sustainable Development (WBCSD) from 2015, and WBCSD from 2021. The contribution of businesses to SD and CSR is exploited according to a complex system of behaviors, and this can be investigated through different perspectives. This paper focuses on CSR communication from a new perspective that aims to obtain insights and measures from Twitter data to understand CSR communication with respect to SDG dimensions.

The set of research questions presented below concerns the topics discussed in the new data source, the CSR Twitter accounts of businesses:

1. How many CSR topics are identified based on our data?

- Which of the three CSR dimensions (social, economic, and environmental) does each topic fall under?
- Which of the SDG dimensions does each topic fall under?

Q2) Are there differences in the relevance of topics across sectors and over time?
Q3) Are there differences in the CSR communication mix across sectors?

With respect to *Q3*, we consider the following hypothesis: CSR dimension weight differs across sectors. Sector differences exist between sectors, which differ by target audience/users and by the characteristics of their products. In particular, these differences are between businesses belonging to the services and businesses providing products, especially final goods.

The innovative contribution of this paper is threefold. First, standing apart from other studies based on survey data, we consider Twitter as our data source and focus on a specific group of companies, namely those included in the Dow Jones Industrial Average index, in order to study their CSR communication and construct indexes. We do not only focus on CSR but also study how the communication relates, in an indirect way, to the SDGs. Second, from the previous discussion (Sect. 2), it is evident that CSR is a dynamic concept linked to different nomenclatures developed over time. For this reason, we do not classify the text into pre-defined categories, but we *let the data speak*. To elaborate, we studied the underlying properties of the text in order to define the topics composing the text and obtain signals from the data. To complete this task, we applied an unsupervised learning technique named *topic modeling*. Specifically, we applied the structural topic model (STM) in order to discover the topics discussed and estimate the differences among sectors and those appearing over time.

Then, starting from the TM results, we propose to compute an innovative index for CSR communication considering different dimensions and use indexes of dissimilarity and homogeneity to identify clusters and study the differences among sectors and firms.

### Data

To carry out our analysis, we selected firms included in the Dow Jones Industrial Average index, a stock market index that measures the performance of the 30 largest listed US companies as of the composition in August 2020. We retrieved the full list of firms, jointly with the activity sector from Bloomberg.^2^ With respect to sector classification, Bloomberg adopts the Global Industry Classification Standard (GICS) developed by MSCI and S&P Dow Jones.^3^ In particular, the firms in our study belong to the following sectors: technology, financial, health care, industrial, consumer discretionary, consumer staples, communication and energy and materials. Next, we identified the official Twitter accounts of the companies. In this respect, it is important to note that each company can have more than one account according to business units, regions, and communication purposes.

We organized the accounts into three categories: (1) Multipurpose business, (2) News-type, and (3) CSR communication accounts (that were general or for specific activities). For the purpose of our analysis, we primarily considered CSR-related accounts in the US. If a CSR account was not available for a company, we selected the news-type accounts, through which firms shared information about company activities. If such an account was not available, we considered the multipurpose one. The underlying reason is that if a CSR-dedicated account is not available, CSR communications are shared through other accounts. We acknowledge that from a statistical point of view, the analysis and selection of Twitter data has some issues. Salvatore et al. (2021) discuss such issues with reference to Twitter data for the construction of social indicators. With reference to our analysis, one is related to the data selection process. Indeed, we gave priority to CSR-specific accounts only when other types were not available. However, after implementing the TM, we focused only on CSR-related topics, discarding the remaining text. Thus, for the purpose of our analysis, such a selection process allows for the reduction of noise in the data with respect to non-CSR tweets.

The full set of original tweets (including retweets without a comment) posted on the firm’s timeline were collected using the Academic Twitter API.^4^ As a reference period, we selected the year of 2019. Indeed, due to the coronavirus outbreak, 2020 represents a special period that is worthy of separate analysis to understand the differences between the communication patterns and topics discussed in the two years. As it happened, Apple and Walgreens Boots Alliance did not have a Twitter account. Thus, the final group included 28 firms, and the total number of accounts we processed was 42: 18 CSR, 5 news-type, and 19 multipurpose business accounts. It was observed that most of the companies operate in the technology sector (21.4%), followed by the financial, health care, and industrial sectors (14.3% each), consumer discretionary and staples (10.7% each), and finally, communication and energy and materials (7.14% each). The total number of messages retrieved is 25,148, with a mean of 2096 messages per month.

Before applying the TM, the text needed to be pre-processed. This phase is also called *data cleaning*, and it is an important step of the analysis, aiming at keeping only relevant words, reducing the complexity of the model, and speeding up the estimation process. Data cleaning involves different operations: the elimination of punctuation, symbols, numbers, stop words, and URLs; conversion of text to lowercase; and stemming. In addition, we removed currencies (for example, $100) and words with less than two characters from the text. We split words with hyphenation and hyphenation-like characters, and we remove company identifiers, including names and Twitter handles (usernames).

After the cleaning process, only relevant terms remained. However, an additional step in data cleaning was the removal of infrequent terms or, in other words, those that appear in a small number of documents. This operation is highly recommended because it allows a researcher to perform noise reduction in the data, making topic detection easier. While it is always important to look at the vocabulary composition, the *rule of thumb* is to remove the terms that appear in less than 0.5–1% of the documents (Denny & Spirling, 2018). Considering the vocabulary and the number of documents removed, and since we were considering tweets composed of a few words (maximum 280 characters), we decided to set this threshold to 0.1%, corresponding to the deletion of terms that occurred in less than 25 documents. Since tweets are short, after the cleaning process, some messages may contain only a few words, thus making it harder to define the topic. For this reason, we considered only documents with more than five words. After these additional data cleaning actions, the resulting final dataset was composed of 22,716 documents and 2029 terms in the vocabulary or features.

## Methods

### Structural Topic Model (STM)

TM is an unsupervised learning technique that allows a researcher to study the underlying properties of a text to discover the topics discussed and obtain signals from the data. Contrary to supervised learning techniques, where researchers classify the text into pre-defined categories and labeled datasets are necessary in order to train the machine and implement the model, in unsupervised methods, such labels are not available and the objective is to understand the latent properties of the text defining each topic. Grimmer and Stewart (2013) provide an extensive review of such methods for text analysis. In this respect, different algorithms are used to implement topic modeling, such as the well-known latent Dirichlet allocation (LDA) (Blei et al., 2003), the bi-term topic model (BTM) (Yan, Guo, Lan, & Cheng, 2013) designed for the analysis of short texts, the latent semantic analysis (LSA) (Dumais, 2004), the non-negative matrix factorization (NMF) (Chen et al., 2019), neural network and deep learning methodologies, and many other models based on different data representation structures—for example, bag of words (BoW) or vector space. Albalawi et al. (2020) provide an extensive review of topic modeling methods for the analysis of short-text data, comparing the aforementioned methods and many others. In their study, LDA and NMF were the best performers.

Recently, in the field of social science, a new topic model has been developed: the STM. It is a variant of the LDA, originally developed to analyze short open-ended survey questions, which, unlike other methods, allows researchers to test hypotheses about document-level metadata on the discussion proportion and word usage of a topic (Roberts et al., 2014, 2016). This model is attuned to our aim of testing our *Q2* hypothesis, which states that topic proportions are significantly different across sectors and over time. It has already been applied in the field of sustainability to study short open-ended questions about climate change (Tvinnereim & Fløttum, 2015) and to SM data^5^ but, as the best of our knowledge, not about sustainability related matters.

STM is a probabilistic mixed membership model that allows researchers to estimate a model that includes document-level metadata and, thus, to study the relationship between topics and metadata. In this section, we briefly describe the model; for further technical details, please refer to Roberts et al. (2016) study, where the model was originally proposed. This model is based on the bag of words (*BoW)* representation, which means that each document is represented as a vector of words without giving importance to the order in which they appear.

**Fig. 1:**
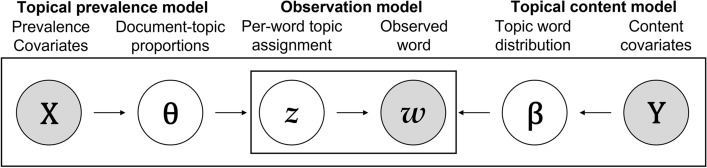
Structural topic model. Source: Amended from Robert, Stewart, & Airoldi’s *(2016)* study

Let us consider a set of $\mathrm{D}$ documents indexed by $\mathrm{d} \in \left\{1,\dots ,D\right\}$. Each document is composed of a mixture of ${N}_{d}$ words ${w}_{d,n}$, where $\mathrm{n} \in \left\{1,\dots {,N}_{d}\right\}$ indicates the position of the word within the document. The collection of unique words is represented by the vocabulary. Each term in the vocabulary is indexed by $\mathrm{v} \in \left\{1,\dots ,V\right\}$, and it is assigned to a topic and associated with the probability of belonging to each topic $\mathrm{k} \in \left\{1,\dots ,K\right\}$. Thus, a topic is a mixture of words, and the document is a mixture of topics. Overall, the model entails three components: a topical prevalence model, controlling how words are allocated to topics as the function of covariates; a topical content model, controlling the frequency of the terms in each topic as a function of the covariates; and an observation model, combining the topic prevalence and topic content models to produce the actual words in each document. Figure 1 summarizes the STM and highlights its three components.

Document metadata are allowed to influence two components of the model: the *topical prevalence,* which is defined as the proportion of the document associated with a topic, and *topical content,* which refers to the usage rate of the word in a topic. Thus, topical prevalence covariates affect the discussion proportion of the topic (θ), while topical content covariates affect the rate of word usage within a topic (β). The matrices of the $\mathrm{P}$ topic prevalence covariates and $\mathrm{A}$ topical content covariates are denoted by ${X}_{D\times P}$ and ${Y}_{D\times A}$, respectively. The model entails the specification of the data generating process for each document, given the number of topics *K*, observed words $\left\{{w}_{d,n}\right\}$, design matrices for topic prevalence *X* and topic content *Y*, and a number of parameters. Covariate information is introduced into the model using generalized linear models. Essentially, prior distributions with globally shared mean parameters in the LDA model are replaced with means parameterized by a linear function of observed covariates. For topic prevalence, a logistic normal distribution is used, with a mean vector parameterized as a function of the covariates. For topical content, an exponential family model is adopted and then parameterized as a function of the marginal frequency of occurrence deviations for each term and of deviations from it that are specific to topics, covariates, and their interactions.

Inference from the model entails the estimation of the posterior distribution. Due to posterior intractability and non-conjugacy, model estimation and inference are based on an approximate variational EM algorithm (Laplace approximation). In the E-step, the variational posterior for each document’s topic proportions is updated, and in the M-step, the topical prevalence and content coefficients are estimated, maximizing the approximated *evidence lower bound* on the marginal likelihood*.* The model converges when the relative change in the approximate variational lower bound is below a defined tolerance level. The model is developed in the R package stm (Roberts, Stewart, & Tingley, 2019), which handles model estimation, summary, and visualization. Further, it is used in the present paper. The tolerance level default value in the R function for the estimation is 10^–5^.

When estimating the model, the analyst must specify the algorithm initialization strategy and the number of topics. A shortcoming of topic models is that the output is very sensitive to initialization. *Spectral initialization*, a deterministic algorithm based on the method of moments, is suggested due to its stability (Roberts, Stewart, & Tingley, 2019). As for choosing the optimal number of topics, the recommendation is to compare five different metrics: held-out likelihood, evidence lower bound, residual dispersion, and semantic coherence along with exclusivity.

The *held-out likelihood* is a measure of predictive power that is useful for model comparison. It can be estimated using the document completion approach (Roberts, Stewart, & Tingley, 2019). The higher the held-out likelihood, the higher the model’s predictive power.

The *evidence lower bound*, which is maximized through the EM algorithm, is also useful in model selection; here, higher values are preferred. Indeed, higher values indicate a better fit of the model to the data.

*Residual dispersion*, a goodness-of-fit measure, was proposed by Taddy (2012), and it builds around estimated *residual dispersion*. Essentially, this method sets up the hypothesis test on residuals’ dispersion, entailing that a large number of topics should be preferred when rejecting the null hypothesis. However, this requirement is very strict, and for practical purposes, it is suggested to look at residual dispersion together with other metrics.

*Semantic coherence* was presented by Mimno et al. (2011) and is calculated for each topic $k$. It provides a measure of the co-appearance rate of the most probable words in that topic. If the most probable words in the topics tend to co-occur, then the topic is semantically coherent. However, if the number of topics is small, they will likely be composed of the same words. In order to overcome this issue, Roberts Stewart, & Airoldi (2016) suggest analysts to consider an *exclusivity* measure that combines the term frequency and term exclusivity to the topic, called FREX (Airoldi & Bischof, 2016). It measures whether the top words for that topic do not appear as top words in other topics. More details about these two metrics are discussed in Appendix 1.

Thus, while choosing the number of topics, researchers should look at all these metrics together. It is important to highlight that this procedure does not yield the *true* number of topics. Rather, it is necessary to validate the choice by manually inspecting the results. To implement the analyses, we used R (R Core Team, 2021). Specifically, for data cleaning, we used quanteda (Benoit, et al., 2018), and for implementing the topic model, we used stm (Roberts, Stewart, & Tingley, 2019).

### Twitter-Based Indexes

The innovative contribution of this paper is the construction of a CSR communication index using the topic model results from the analysis of Twitter data. The study of social media communication together with traditional indexes can help in better understanding how firms are addressing sustainability in their corporate strategy. This index measures the overall proportion of corpora related to a CSR dimension. The dimension considered are linked to the three SDG pillars, namely economic, social and environment, as discussed in Sect. 2.

In order to study the Twitter-based CSR communication mix, we considered the TM results—that is, the proportion of text that belongs to each topic. Then, we assigned topics to CSR dimensions to obtain the proportion of text for each. These proportions are aggregated at the sector level. In addition, we propose to use dissimilarity and homogeneity indexes to identify sectors or companies that put their effort toward a specific dimension and clusters of sectors or companies showing similar behavior toward CSR communication. Such indexes are commonly used in the literature related to economics, political science, ecology, and social science in order to study differences across individuals and the polarization of preferences and behaviors (Piccolo, 2010).

To build the index, first, we assigned topics to one of the CSR dimensions—namely economic (Eco), environment (Env), and social (Soc). Given the specific characteristics of communication on Twitter, when addressing *Q1* about which topics are discussed and how they relate to CSR and SDGs, we also considered a fourth dimension: the mixed and general CSR dimension (Mix). This is because the twitter communication is characterized by audience involvement and a specific wording which makes difficult to assign some topics only to one dimension. It is described in more detail in Sect. 5.2. Topics that are not related to CSR are not considered in the analysis. Second, for each tweet, we summed the proportion of topics belonging to the same dimensions. Then, we aggregated the results at the firm and sector levels to obtain dimension indexes.

The resulting dimension indexes are consistent with the TM philosophy, which assumes that each text is composed of multiple topics. Indeed, they express the overall proportion of the corpora that belong to a dimension, allowing tweets to be composed of a mix of topics (*soft assignment*). These are also frequency distributions and, in order to distinguish between firms or sectors, which give the same importance to all dimensions or which are specialized in one of them, it is important to study their heterogeneity. For this purpose, given a frequency distribution over CSR dimensions, ${f}_{i}^{(j)}$, *i* = *1, 2, 3, 4* denoting CSR dimensions and *j* = *1, …, J* denoting sectors, we proposed to compute the Frosini heterogeneity index for each sector as follows (Piccolo, 2010):

1 $$F_{j} = 1 - \sqrt {\frac{4}{4 - 1} \cdot \mathop \sum \limits_{i = 1}^{4} \left( {f_{i}^{\left( j \right)} - \frac{1}{4}} \right)^{2} } , j = 1, \ldots ,J.$$

This index is bounded between 0 and 1. Further, $${F}_{j}=0$$ represents the case of *minimum heterogeneity* (the communication is concentrated on one dimension only), while $${F}_{j}=1$$ corresponds to *maximum heterogeneity* where the communication is equally split over all dimensions (namely, the dimensions’ frequencies are all $$1/4)$$. The literature suggests several measures of heterogeneity, such as the Gini and Shannon indexes. Such popular indexes, although normalized, vary in a small range and do not allow for effective discrimination. On the contrary, the Frosini index discriminates better than the Gini and Shannon indexes and is preferred for this reason (Piccolo, 2010).

To further analyze the composition, we considered the following relative dissimilarity index between each couple of CSR dimension frequency distributions (Piccolo, 2010). Given two CSR dimensions relative frequency distributions for sectors *j* and *l*, denoted by ${f}_{i}^{(j)}$ and ${f}_{i}^{(l)}$, the dissimilarity index is defined as follows:

2 $$d_{jl} = \frac{1}{2} \cdot \mathop \sum \limits_{i = 1}^{4} \left| {f_{i}^{\left( j \right)} - f_{i}^{\left( l \right)} } \right|, j,l = 1, \ldots ,J.$$

This relative dissimilarity index is bounded between 0 and 1. When ${d}_{jl}=0$, the communication composition for sectors *i* and *j* is exactly the same. In this case, communication composition for the two sectors is said to be similar (*minimum dissimilarity*). On the contrary, when ${d}_{jl}=1$, the communication for one sector is concentrated on one dimension say, (${f}_{i}^{(j)}=1 \text{for some}\, i)$, while the communication for the other sector is concentrated on a different dimension (${f}_{m}^{(l)}=1, \text{for some}\, m\ne \mathrm{i})$. This is the case of *maximum dissimilarity*. By constructing a dissimilarity matrix, it is possible to compare the communication composition among all sectors and identify clusters. This allows us to highlight differences among sectors in the communication of CSR activities. Similar indexes can be defined at the firm level.

## Results and Discussion

### Model Selection

After the cleaning phase, the resulting dataset is composed of 22,716 documents and 2029 vocabulary terms; these data are ready to be analyzed. At first, the model must be specified, and the number of topics identified following the methodology presented in Sect. 4.1. In our analysis, we only included topic prevalence covariates: sector and week. To process the data, for each tweet we consider the week it was published. This time dimension is considered to study the temporal evolution of topics. That is because it provides a good number of details for event detection and assures a good amount of data for each period. Thus, we allowed sectors and weeks to affect the discussion proportion of a topic. We estimated the effect of the week variable through splines to account for non-linear effects. Next, in order to choose the number of topics, we proceeded with the computation of the five metrics discussed in Sect. 4.1—namely, (1) Held-out likelihood, (2) Lower bound, (3) Residuals, (4) Semantic coherence, and (5) Exclusivity.

**Fig. 2:**
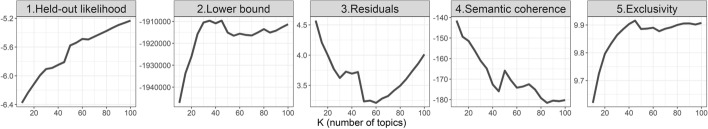
Evaluation metrics for choosing the number of topics. Source: Authors’ own elaboration

Figure 2 shows the five metrics for different numbers of topics, from 10 to 100, with a step size of 5. Looking at the picture, the trade-off between semantic coherence and exclusivity is evident. High values of all metrics should be preferred—except for residuals, which should be minimized. To summarize the graphical analysis, Table 1 shows the ranges of topics where such metrics were good. This corresponds approximately to a number of topics between 45 and 50. It should be clear that these metrics only offer an indication and that there is no fixed way to choose the right value for $K$ since this procedure does not yield the *true* number of topics. Thus, in order to select an appropriate number, it is necessary to manually inspect the results. Given the indication from the comparison of the five metrics, we focused on a number of topics between 45 and 50, and after having manually inspected the results (topic composition), we concluded that the model with 47 topics seemed to be the most appropriate, providing a good number of topic details. Table 7 provides the values of the five metrics for *K* between 45 and 50.

**Table 1:** Model selection metrics and range of plausible number of topics: + indicates that higher values should be preferred and – indicates the opposite. Source: Authors’ own elaboration

Metric	Criterium	Range of topics
Held-out likelihood	+	> 45
Lower bound	+	30–50
Residuals	–	45–60
Semantic coherence	+	45–55
Exclusivity	+	45–55

### Topic Discovery

Topic discovery is performed by looking at the most probable words for each topic and tweets with a highs topic proportion and then by labeling them accordingly. The complete results are reported in Appendix 3 and in Tables 8, 9, and 10, where, for each topic, the top seven stems according to different metrics are reported. The majority of topics (20) relates to social aspects, including enabling inclusivity, equality, and well-being; supporting small businesses and communities; organizing educational programs; and making cities safer and equitable to give birth and raise children.

Next, economic aspects are discussed (six topics), such as announcements about partnerships, information on financial and economic performances, and CEO communications. Two topics concern environmental issues, such as clean water, waste management, pollution, and clean energy. Interestingly, we also identified eight topics that are not about a specific dimension but show mixed communication on specific and general CSR activities. Thus, we considered an additional fourth dimension. These topics, reported in Table 9, emphasize the multidimensional characteristics of CSR, with businesses extending along the dimensions of philanthropy and voluntarism in their communication with stakeholders as well. Thus, in their transition to sustainability, businesses are committed to the information and sponsorship of cultural/sports events as well as to making clear how their products are in line with SD. These topics are peculiar because they refer to a type of communication aiming to boost audience involvement through the sponsorship of real and online events or because they invite the audience to read blog posts about business initiatives. We categorize these topics under the dimension “mixed and general CSR communication”. Finally, 11 topics do not relate to CSR but are product or service advertisements (Table 10). This topic supports two aspects of CSR communication. The first one is that sometimes, through SM communication, the promotion of products is also handled. The second is that in some cases, each of the three dimensions of CSR (pillars for SDGs) is not clearly identified in the behavior of the interaction with stakeholders.

Looking at the topic composition, we can relate SM communication to SDGs. We do that only for the CSR topics that are not mixed. Table 2 generally describes the topics related to each SDG and CSR dimension. SDGs are interrelated, and in the table, we consider only the primary SDGs. We then discuss the relationship between SDGs. From the analysis of topics, it is evident that businesses are involved in several activities to promote SD, which are not merely philanthropic or linked to their type of activities but also truly have an impact on society and the global community.

**Table 2:** Description of SDG and CSR topics. Source: Authors’ own elaboration

SDG	CSR Dimension	No. of Topics	Description of topics
SDG 8: Decent work and economic growth	Economic Social	6 1	CEO talks about leadership Economic impact of the business Announcement of partnerships Workplace well-being
SDG 9: Industry, Innovation, and Infrastructure	Social	4	Social impacts of innovation and digitalization Human–machine interaction to improve everyday life
SDG 10: Reduced inequalities	Social	4	Accessibility and inclusiveness (disability) Creating a better world for everyone Fighting racial discrimination Closing the gaps (in gender, digital spaces, and other areas)
SDG 11: Sustainable cities and communities	Social	4	Preserving the culture of communities Sustaining small businesses Celebrating and supporting communities Safer and more equitable cities
SDG 3: Good health and well-being	Social	2	Promoting the importance of scientific research for well-being, vaccinations, and disease prevention
SDG 4: Quality education	Social	2	Grants and scholarship for students Encourage STEM education
SDG 5: Gender equality	Social	2	Gender pay gap Women in business and STEM Women’s empowerment
SDG 1: No poverty	Social	1	Business actions to fight extreme poverty
SDG 6: Clean water and sanitation	Environment	1	Clean water to all global communities World Water Day Oceans and Marine Conservation Water pollution
SDG 7: Affordable and clean energy	Environment	1	Initiatives to promote clean energy and reduce pollution

While primary SDG goals have been identified and linked to topics, it is clear that the goals are related to each other. For example, SDG 10 (reduced inequalities) is naturally linked with the theme of gender equality (SDG 5) and the reduction of inequalities within and between countries (SDG 1: no poverty). Similarly, when gender equality relates to initiatives improving workplace and working conditions, then it is also linked to SDG 8 (decent work and economic growth). Additionally, SDG 4 (quality education) can be linked to SDG 11 (sustainable cities and communities). Topic 5, which is about the promotion of grants for schools and students, also relates to grants released and competitions held for the preservation of historical buildings and community cultures.

**Fig. 3:**
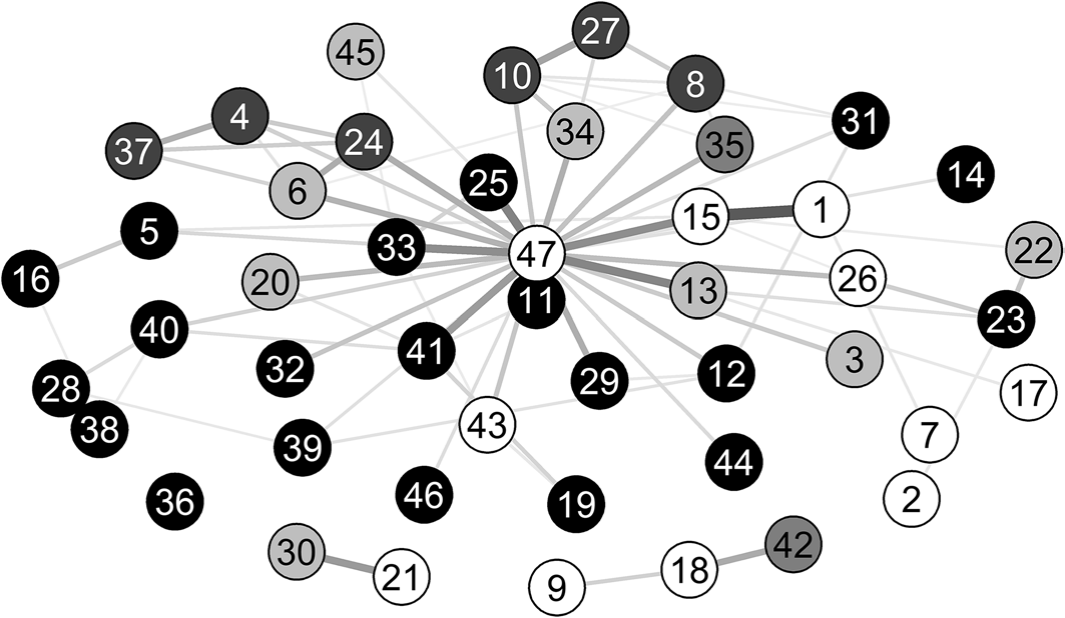
Topic correlation: black for social topics, dark grey for economic topics, grey for environmental topics, light grey for mixed and general CSR topics, and white for non-CSR topics. Source: Authors’ own elaboration

The STM model allows topics to be correlated. Figure 3 represents the topic correlation network, showing positively correlated topics—that is, topics that are likely to be discussed together within a tweet. Correlations within the same dimensions are evident. It is interesting to see how Topic 47, which is made up of general words, is central and correlated to the majority of topics.

With respect to the first research question *Q1*, the analysis of the STM model allowed to understand the online communication about CSR and SDGs. In particular, a fourth CSR dimension, namely Mixed-General CSR, was triggered in order to fully characterize the online communication through Twitter. The relationship between CSR and SDGs have been highlighted, showing that they are two interrelated phenomena.

### Covariate Effect

The characterizing feature of this model is the possibility of estimating the effects of topical prevalence covariates on the discussion proportion of a topic.

As expected, some sectors talk more about certain topics. Indeed, looking at the estimated topic proportion and the 95% confidence interval, it is evident that firms in the health care sector tweet more about well-being and health issues (Topics 28 and 38). Topics about human–machine interactions, the social impact of technology, and the closure of the digital divide are discussed mainly by businesses in the technology and communication sectors (Topic 19, 23, 25, and 33). Then, firms in the financial, energy and materials, and industrial sectors talk more about economic topics. Firms in the energy and materials sector also talk about environmental issues, especially with reference to clean energy and pollution. Finally, there are mixed and general CSR topics. The majority of these topics is linked to the sponsorship of events, and for that reason, we see peaks in the estimated topic proportion over time.

**Fig. 4:**
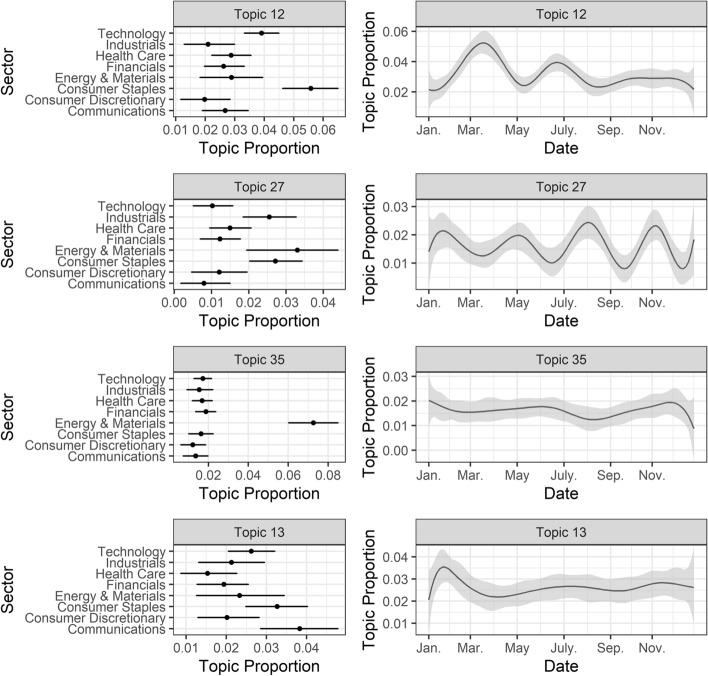
Effect of the “sector” and “time” on the proportion of the topics discussion. Source: Authors’ own elaboration

Hereafter, we discuss the results in detail for only one topic by dimension (Fig. 4). The full results for all topics are shown in Appendix 4 (Figure 7, 8, 9, 10, 11, 12, 13, 14). Topic 12 is about the social dimension, and it is related to gender equality and, in particular, the pay gap and women’s empowerment with the promotion of specific awareness events organized for the International Women’s Day (examples of hashtags: #IWD2019, #GenderEquality, #WomensHistoryMonth). That topic shows a higher proportion for firms in the consumer staples and technology sectors, whose companies promoted and sponsored initiatives for gender equality. Moreover, looking at the estimated topic proportion over time, there is a peak in the discussion in March, when International Women’s Day is celebrated, and in June, when the Women Deliver conference (for gender equality and the health, rights, and well-being of women) took place.

Topic 35 relates to business reports or CEO talks about sustainability initiatives that promote clean energy and reduce emissions and pollution, and it is quite stable over time. This is specific to the energy and materials sector. It should be noted that the climate question is also part of Topic 46 as a global issue, in addition to poverty. The second topic about the environment needs clarification. It is Topic 42 about clean water, World Water Day, ocean and marine conservation, and water pollution. However, due to the wording, the subjects of insurance services against weather conditions and household problems (burst pipes) are also prevalent in tweets. Finally, for the mixed and general CSR category, we considered Topic 13, which is about the sponsorship of events—particularly the Super Bowl that took place in February. Indeed, we can see that the topic proportion peaks during this period. It was sponsored by several companies, and for that reason, there is no clear distinction between sectors.

This analysis allowed us to answer *Q2*. Indeed, the STM proved to be useful in order to study differences in topical prevalence across sectors and over time. It allowed to get signal from the data and distinguish between sector and time specific topics.

### CSR Twitter Indexes

**Fig. 5:**
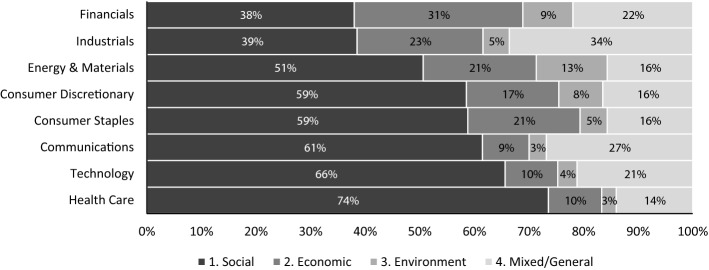
Communication mix by sector. Source: Authors’ own elaboration

The aim of this study is to get insight into the communication mix of firms and sectors. To this purpose, we analyse the twitter CSR dimension indexes together with those proposed in Sect. 4, in order to identify clusters and dimension-specific sectors. Figure 5 shows the communication mix across sectors.

It is evident that some sectors are specific to dimensions. The health care sector mainly focuses on the social dimension with a percentage equal to about 74%. The quote of social communication is higher than 50% for all other sectors, excluding financial and industrial ones. Economic communication is specific to the financial sector, which has the highest proportion, equal to 31%. The economic dimension has a proportion of around 20% in all other sectors, except communications, technology, and health care (10% on average). Environmental communication is more important in the energy and materials, finance, and consumer discretionary sectors, while in the other sectors, it is marginal (lower than 5%). Mixed and general CSR communication is the prevalent dimension in the industrial sectors with a share of about 34%, followed by the communication sector with a percentage of 27%.

In order to identify clusters and compare sectors, we computed the Frosini heterogeneity index and the dissimilarity index between each couple of frequency distributions, as discussed in Sect. 4.2. The Frosini index allows researchers to assess the heterogeneity of distributions in order to identify sectors that specialize in one dimension. A value of the index equal to zero corresponds to minimum heterogeneity, while a value of one corresponds to maximum heterogeneity. Table 3 ranks the sectors based on the corresponding Frosini index computed according to Eq. (1). Table 4 shows the dissimilarity index for sectors (Eq. (2)). It is bounded between 0 (minimum dissimilarity) and 1 (maximum dissimilarity).

**Table 3:** Frosini index at the sector level. Source: Authors’ own elaboration

Sector	Frosini Index
Financials	0.75
Industrials	0.70
Energy and materials	0.65
Consumer discretionary	0.55
Consumer staples	0.53
Communications	0.47
Technology	0.44
Health care	0.35

**Table 4:** Dissimilarity matrix at the sector level: light grey for low dissimilarity and dark grey for high dissimilarity. Source: Authors’ own elaboration

No. of tweets	Sector	COM	CD	CS	E&M	FIN	HC	IND	TECH
1667	COM	0.00							
1450	CD	0.13	0.00						
1980	CS	0.14	0.04	0.00					
571	E&M	0.22	0.09	0.08	0.00				
3943	FIN	0.28	0.21	0.21	0.16	0.00			
3092	HC	0.13	0.15	0.15	0.23	0.36	0.00		
1462	IND	0.23	0.23	0.20	0.20	0.12	0.35	0.00	
8551	TECH	0.06	0.12	0.12	0.21	0.28	0.08	0.27	0.00

Considering the Frosini indexes together with the frequency distributions in Fig. 5, it is possible to identify dimension-specific clusters. The first is composed of the communication, technology, and health care sectors, where the Frosini and dissimilarity indexes are generally lower compared to other sectors. Further, the prevalent dimension is the social one, followed by the mixed and general one, indicating a communication effort made to boost audience involvement. For the consumer discretionary and staples sector, social communication is still the most prevalent dimension. However, the economic one also gains importance. In contrast, the three remaining sectors present a wider communication mix corresponding to a higher Frosini index. Specifically, communication is more heterogeneous in the financial sector, while it is more homogeneous in the health care sector, where the social dimension prevails. The same indexes can also be computed at the firm level, exercising caution in our interpretation for firms with a relatively low number of tweets. In this respect, we also restricted the analysis to firms with more than 100 tweets.

**Fig. 6:**
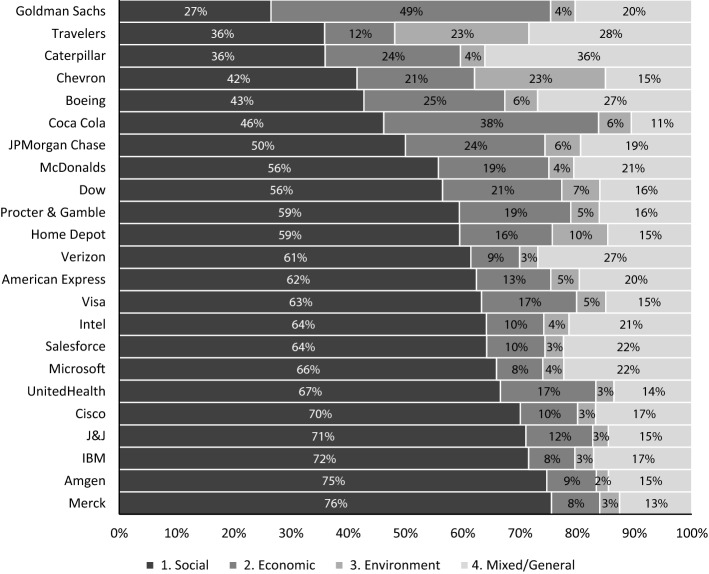
Communication mix by firms. Source: Authors’ own elaboration

Looking at the frequency distributions (Fig. 6), the Frosini index (Table 5), and the dissimilarity matrix (Table 6), the detailed analysis of firms highlights that, in general, the results are similar to those at sector levels, but some differences within the same sector are present. A special case is the financial sector, where American Express and Goldman Sachs have different behaviors. The former shows a communication style that is more similar to the health care and technology sectors, which clearly prefer social communication (Fig. 6). The latter is an interesting case since it differs from most of the other firms (Table 6), strongly preferring economic communication. Furthermore, Caterpillar is a firm with more mixed and general communication aiming to boost audience involvement. Besides, Chevron (from the energy sector) and Travelers (from the financial sector) talk about environmental issues. Indeed, following the previous discussion about topic discovery, we found that Topic 42 (environment) is also prevalent in tweets about insurance services against weather conditions or household problems (burst pipes) due to similar wording. Then, there are sectors in which firms’ behavior is almost the same, such as technology and health care.

**Table 5:** Frosini index at the firm level. Source: Authors’ own elaboration

Firm	Sector	Frosini Index
Travelers	Financials	0.80
Chevron	Energy and materials	0.77
Caterpillar	Industrials	0.70
Boeing	Industrials	0.70
JPMorgan Chase	Financials	0.63
Goldman Sachs	Financials	0.63
Coca-Cola	Consumer staples	0.60
McDonalds	Consumer discretionary	0.56
Dow	Energy and materials	0.56
Home Depot	Consumer discretionary	0.54
Procter & Gamble	Consumer staples	0.52
American Express	Financials	0.49
Visa	Technology	0.48
Verizon	Communication	0.47
Intel	Technology	0.46
Salesforce	Technology	0.45
UnitedHealth	Health care	0.43
Microsoft	Technology	0.43
Cisco	Technology	0.39
J&J	Health care	0.38
IBM	Technology	0.37
Amgen	Health care	0.33
Merck	Health care	0.32

**Table 6:** Dissimilarity matrix at the firm level: light grey for low dissimilarity and dark grey for high dissimilarity. Source: Authors’ own elaboration

No. of tweets	/	VZ	HD	MCD	KO	PG	CVX	DOW	AXP	GS	JPM	TRA	AMGN	JNJ	MRK	UNH	BA	CAT	CSCO	IBM	INTC	MSFT	MRC	V
1640	**VZ**	0.00																						
1158	**HD**	0.14	0.00																					
273	**MCD**	0.12	0.09	0.00																				
139	**KO**	0.32	0.21	0.20	0.00																			
1770	**PG**	0.13	0.05	0.04	0.19	0.00																		
225	**CVX**	0.32	0.18	0.20	0.22	0.19	0.00																	
346	**DOW**	0.16	0.06	0.05	0.17	0.03	0.16	0.00																
712	**AXP**	0.07	0.08	0.07	0.25	0.06	0.25	0.09	0.00															
1436	**GS**	0.41	0.38	0.30	0.21	0.34	0.34	0.32	0.37	0.00														
831	**JPM**	0.19	0.13	0.07	0.13	0.09	0.17	0.07	0.13	0.25	0.00													
964	**TRA**	0.26	0.28	0.27	0.36	0.31	0.14	0.29	0.27	0.37	0.26	0.00												
1390	**AMGN**	0.13	0.15	0.19	0.32	0.15	0.33	0.18	0.12	0.48	0.25	0.39	0.00											
768	**JNJ**	0.13	0.12	0.15	0.29	0.12	0.30	0.15	0.09	0.45	0.21	0.35	0.04	0.00										
770	**MRK**	0.14	0.16	0.20	0.31	0.16	0.34	0.19	0.13	0.49	0.26	0.40	0.02	0.05	0.00									
164	**UNH**	0.13	0.08	0.11	0.23	0.07	0.25	0.10	0.08	0.40	0.17	0.35	0.09	0.05	0.09	0.00								
163	**BA**	0.19	0.21	0.13	0.16	0.17	0.17	0.15	0.20	0.24	0.08	0.19	0.32	0.28	0.33	0.24	0.00							
1201	**CAT**	0.25	0.29	0.20	0.26	0.24	0.24	0.23	0.27	0.25	0.17	0.19	0.39	0.35	0.40	0.31	0.09	0.00						
927	**CSCO**	0.10	0.13	0.14	0.30	0.11	0.30	0.14	0.08	0.44	0.20	0.34	0.05	0.03	0.06	0.07	0.27	0.34	0.00					
230	**IBM**	0.10	0.15	0.16	0.32	0.13	0.32	0.16	0.09	0.45	0.22	0.36	0.04	0.04	0.05	0.09	0.29	0.36	0.02	0.00				
666	**INTC**	0.05	0.11	0.09	0.29	0.10	0.29	0.13	0.04	0.39	0.16	0.28	0.11	0.09	0.11	0.09	0.21	0.28	0.06	0.07	0.00			
2930	**MSFT**	0.05	0.14	0.12	0.31	0.13	0.32	0.16	0.06	0.41	0.19	0.30	0.09	0.09	0.10	0.09	0.23	0.30	0.06	0.06	0.03	0.00		
3350	**MRC**	0.05	0.12	0.10	0.30	0.11	0.30	0.14	0.05	0.40	0.17	0.28	0.10	0.08	0.12	0.09	0.21	0.28	0.06	0.07	0.01	0.02	0.00	
448	**V**	0.12	0.05	0.08	0.22	0.04	0.22	0.07	0.05	0.38	0.13	0.32	0.11	0.08	0.12	0.03	0.21	0.28	0.09	0.10	0.07	0.10	0.08	0.00

With reference to *Q3* about differences in the communication mix and especially the hypothesis that the communication mix differs between businesses providing services and products, we obtain evidence of this difference. Indeed, firms in the communication, health care, and technology sectors focus more on the social dimension with respect to others.

## Conclusion

SDGs require global actions and efforts from the national and local governments, civil society, and corporations to guarantee the protection of the planet and the progress of society. The role of companies is especially important to address key issues through the development of new business models that positively impact local and global communities. This is related to the contemporary view of CSR activities, according to which CSR is part of daily business life. To fully implement CSR firms should engage in activities to promote social good beyond economic interests. Moreover, the importance of stakeholders and their influence are now recognized. Thus, the management of the relationship between companies and stakeholders is crucial to improve the path of society toward sustainability. SM could be an effective tool for CSR communication. The information emerging is particularly interesting and helps one understand the behavior of firms with respect to the 2030 Agenda and the characteristics of their online communication. However, to our knowledge, there are no extensive studies about the characteristics of CSR communication on SM, especially in relation to SDGs. This paper explores CSR communication from this new perspective which refers to the theoretical framework described above. In particular, we addressed three research questions: (1) How the topics discussed in the Social Media Twitter relate to CSR and SDGs, (2) How the topics vary across sectors and over time, and (3) Whether sector behavior with respect to the CSR communication mix is different. From the methodological point of view, the paper is innovative since the construction of CSR indexes is based on TM results.

With reference to the first and second research questions, TM analysis has been applied to identify the emerging topics in terms of CSR dimensions and SDGs and the effect of sector and time on topic proportion. Moreover, original sectoral indexes based on TM results and heterogeneity indexes are proposed and computed.

From the substantive point of view, we found evidence for, besides classic economic and financial reporting, firms being committed to the social dimension of CSR, mainly to support communities with programs to reduce inequalities and discrimination, support local firms, and organize educational programs. Furthermore, environmental issues are relevant and prevalent in the energy and materials sector. Online communication also entails the promotion of online or real events. Then, we identified a fourth CSR dimension—namely, mixed, and general CSR communication—characterized by specific wordings aiming to boost audience involvement. Using the STM model, it was also possible to identify time- and sector-specific topics. Indeed, the model proved to be useful for detecting events and obtaining insights from the data. The analysis also demonstrated that in their sustainability path, businesses align themselves to SDGs and actively communicate about their initiatives online to involve stakeholders.

Based on topic proportions, we studied the communication mix, providing indexes of heterogeneity and dissimilarity to address the third research question. This analysis allowed us to identify clusters and differences among sectors. In particular, our initial hypothesis about the difference in the communication mix between firms that provide services and products has been verified. The social dimension was found to be prevalent in firms providing services (communication, health care, and technology) rather than in firms providing final products.

This study also has some limitations. First, the construction of indexes requires one to assume that the topic is truly linked to that CSR dimension. Second, many choices in topic modeling are left to the analyst’s judgement, including the pre-processing steps and the choice of the number of topics. The impact of text pre-processing techniques on the output of the final analysis has been investigated with reference to supervised machine-learning classification algorithms and unsupervised learning (LDA), but not with reference to the STM that also includes document-level metadata in the estimation. We will address this issue in a forthcoming study. Moreover, when analyzing Twitter data, practitioners should be aware of data quality aspects and the errors they may encounter (Salvatore et al., 2021). As for future developments, topic modeling results can be manually validated and used as a training set to analyze higher volumes of data with supervised classification algorithms in order to label such messages with the corresponding CSR dimension.

Finally, this study also aims to contribute to the growing research on the use of SM data as an innovative data source for the production of new economic statistics and indicators. Such innovative indexes and analyses highlight the behavior of businesses toward the SD framework (including SDGs) and can help policymakers understand the role of businesses with respect to the 2030 Agenda The use of TM has shown interesting performances, this contributes to follow up a promising research line based on text analysis.

## Figures and Tables

**Fig. 7:**
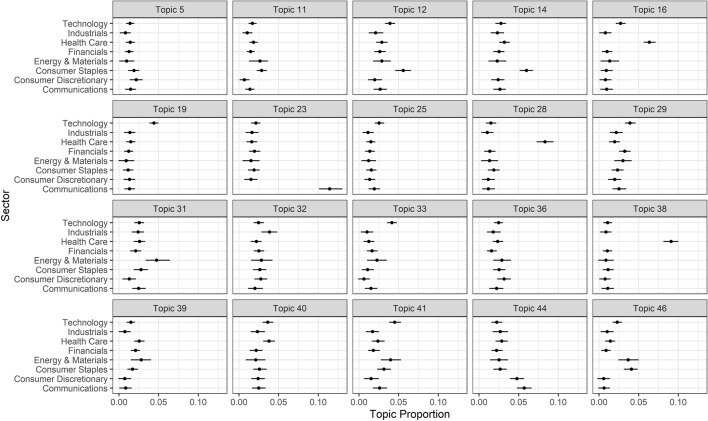
Effect of the “sector” on the proportion of Social topics discussion. Source: Authors’ own elaboration

**Fig. 8:**
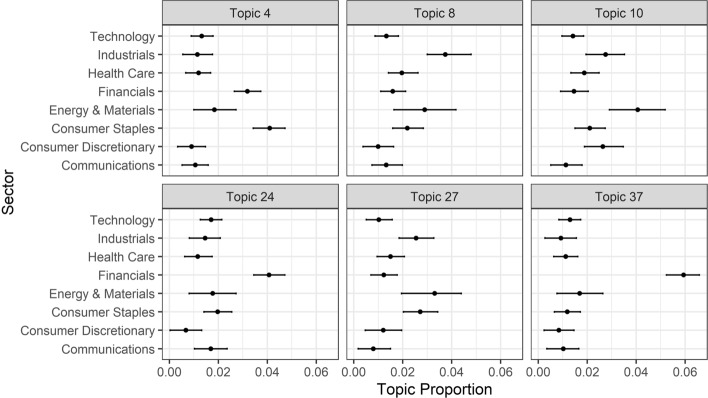
Effect of the “sector” on the proportion of Economic topics discussion. Source: Authors’ own elaboration

**Fig. 9:**
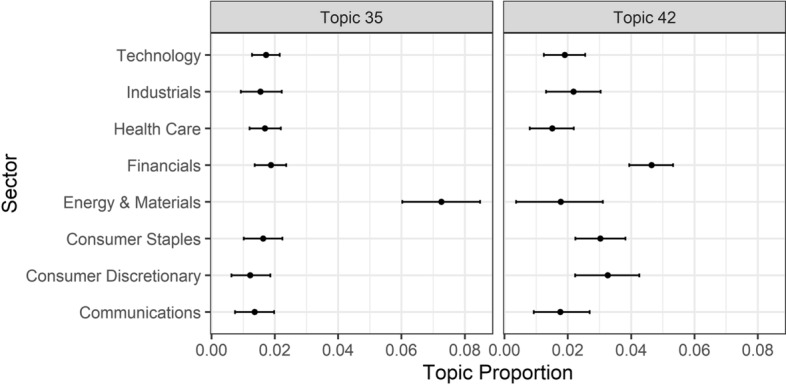
Effect of the “sector” on the proportion of Environmental topics discussion. Source: Authors’ own elaboration

**Fig. 10:**
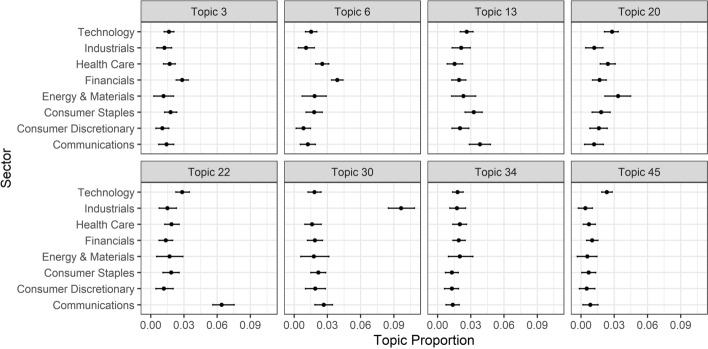
Effect of the “sector” on the proportion of Mixed topics discussion. Source: Authors’ own elaboration

**Fig. 11:**
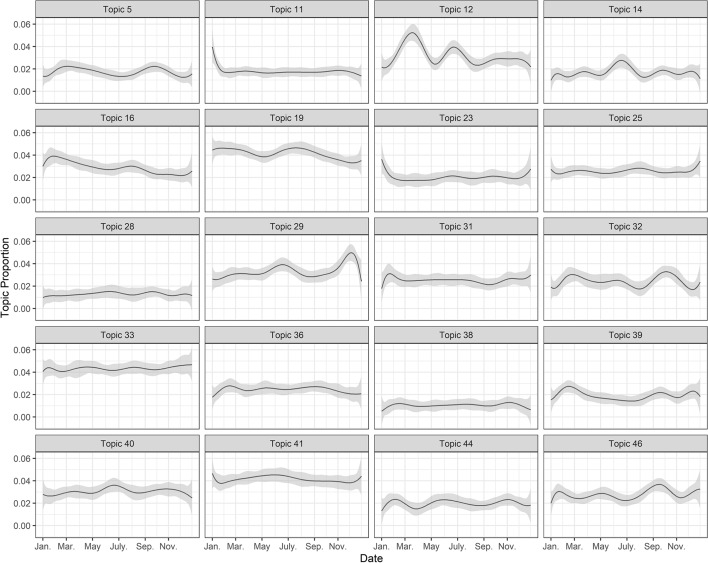
Expected Social topics proportion over time. Source: Authors’ own elaboration

**Fig. 12:**
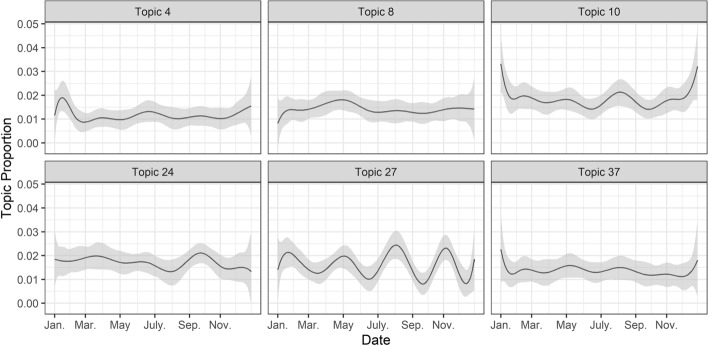
Expected Economic topics proportion over time. Source: Authors’ own elaboration

**Fig. 13:**
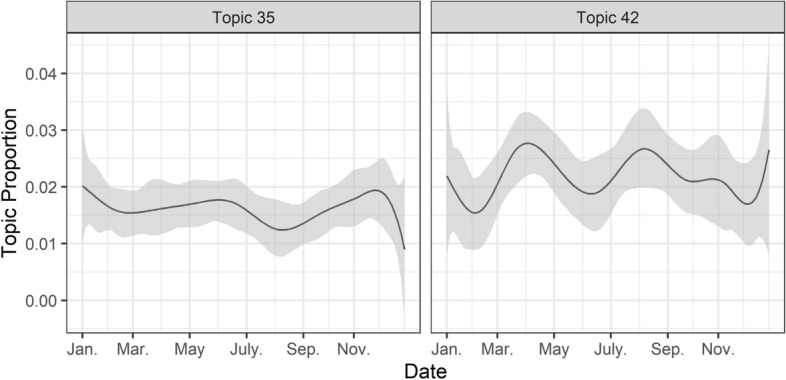
Expected Environmental topics proportion over time. Source: Authors’ own elaboration

**Fig. 14:**
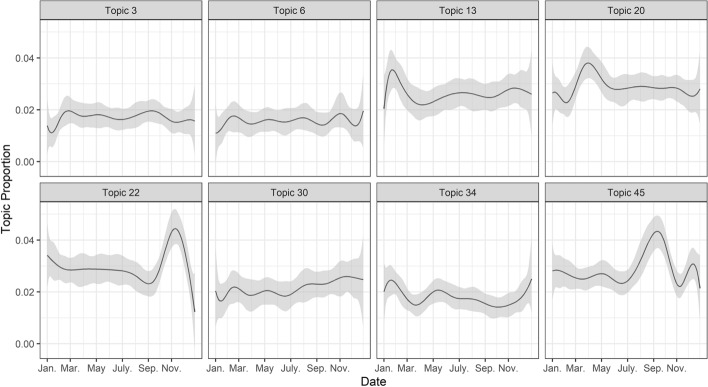
Expected Mixed topics proportion over time. Source: Authors’ own elaboration

## Appendix 1: Semantic Coherence and FREX

Let ${V}^{\left(k\right)}=\left({v}_{1}^{\left(k\right)},\dots ,{v}_{M}^{\left(k\right)}\right)$ be the list of the $\mathrm{M}$ most probable words in topic $\mathrm{k}$. Then, define $\mathrm{D}\left(\mathrm{v}\right)$ as the document frequency for word $\mathrm{v}$ and $\mathrm{D}\left({v}_{m},{v}_{l}\right)$ as the co-document frequency for words ${v}_{m}$ and ${v}_{l}$, i.e., the number of documents in which the selected terms occur together. Then, for each topic ${\text{k}}$, the semantic coherence is defined as follows:

$$C\left( {k;V^{{\left\{ {\left( k \right)} \right\}}} } \right) = \mathop \sum \limits_{m = 2}^{M} \mathop \sum \limits_{l = 1}^{m - 1} \log \frac{{D\left( {v_{m}^{\left( k \right)} ,v_{l}^{\left( k \right)} } \right) + 1}}{{D\left( {v_{l}^{\left( k \right)} } \right)}}.$$

Define $\mathrm{B}\left({v}^{\left(k\right)}\right)$ as the occurrence rate of a word $\mathrm{v}$ in topic $\mathrm{k}$. Then, for a set of comparison topics $\mathrm{S}$, the exclusivity is defined as follows $\mathrm{E}\left(k;v\right)={B\left({v}^{\left(k\right)}\right)}/{{\sum }_{h\in S}B\left({v}^{\left(h\right)}\right)}$. The FREX is defined for each topic $\mathrm{k}$ and term $\mathrm{v}$ as the weighted harmonic mean of term’s frequency and exclusivity:

$$FREX_{k,v} = \left( {\frac{w}{{ECDF\left( {} \right)\left( {{\raise0.5ex\hbox{$\scriptstyle {B\left( {v^{\left( k \right)} } \right)}$} \kern-0.1em/\kern-0.15em \lower0.25ex\hbox{$\scriptstyle {\mathop \sum \nolimits_{h \in S} B\left( {v^{\left( h \right)} } \right)}$}}} \right)}} + \frac{1 - w}{{ECDF\left( {B\left( {v^{\left( k \right)} } \right)} \right)}}} \right)^{ - 1}$$

where ${\text{w}}$ is the weight in favour of exclusivity and ECDF stands for empirical cumulative distribution function.

## Appendix 2: Selection of the Optimal Number of Topics

See Table 7.

**Table 7:** Metrics to select the optimal number of topics. The three best metrics are in bold. Source: Authors’ own elaboration

Topics	1.Held-out likelihood	2.Lower bound	3.Residuals	4.Semantic coherence	5.Exclusivity
45	−5,809	−1,909,647	3,722	−175,936	9,916
46	−5,838	−1,907,921	3,802	−176,545	9,914
47	**−5,608**	**-1,915,321**	**3,256**	−166,924	9,878
48	**−5,602**	**−1,915,063**	**3,250**	−167,480	9,882
49	−5,736	−1,908,061	3,596	−179,731	9,919
50	**−5,577**	**−1,915,064**	**3,235**	−165,889	9,886

## Appendix 3: Topics Composition

Probability (*Prob* in the table) indicates the most probable words inferred by the topic word distribution ($\upbeta$) from the model. The FREX score indicates the stems that are both frequent and exclusive, and, thus, distinguishing topics. We can also look at the expected proportion, which indicates the portion of the overall text assigned to topics, estimated according to the implemented model.

See Tables 8, 9 and 10.

**Table 8:** CSR topics proportion, word composition metrics and CSR/SDG labels. The data are ordered by the Expected topic proportion. Source: Authors’ own elaboration

Topic	Exp. Prop	Top 7 stems	CSR/SDG
41	0.035	Prob: make, world, inclus, access, around, creat, everyon FREX: make, differ, inclus, everyon, around, world, sure	Social Goal 10 Reduced Inequalities
16	0.031	Prob: student, program, educ, scienc, learn, #stem, teacher FREX: student, #stem, teacher, #amgenscholar, #biotechexperi, scienc, code	Social Goal 4 Quality Education
33	0.030	Prob: use, technolog, data, #ai, can, power, learn FREX: #ai, data, technolog, intellig, use, comput, driven	Social Goal 9 Industry, Innovation and Infrastructure
31	0.029	Prob: compani, proud, employe, honor, best, list, top FREX: list, recogn, honor, proud, name, congratul, compani	Social Goal 8 Decent work and economic growth
44	0.028	Prob: first, thank, team, serv, time, veteran, amaz FREX: first, serv, veteran, respond, kind, volunt, amaz	Social Goal 11 Sustainable cities and communities
46	0.027	Prob: global, challeng, solut, problem, take, world, impact FREX: problem, solv, solver, global, poverti, challeng, #activ	Social Goal 1 No poverty
19	0.027	Prob: featur, regist, check, learn, #access, webinar, new FREX: webinar, featur, regist, content, lightn, #access, reader	Social Goal 9 Industry, Innovation and Infrastructure
12	0.027	Prob: women, equal, work, gender, chang, togeth, can FREX: equal, #iwd2019, women, gender, #weseeequ, believ, yes	Social Goal 5 Gender Equality
42	0.026	Prob: can, home, help, water, protect, thing, clean FREX: water, consid, home, drink, clean, fuel, track	Environment Goal 6 Clean water
10	0.025	Prob: year, last, announc, increas, goal, million, billion FREX: year, increas, billion, last, ago, cash, goal	Economic Goal 8 Decent work and economic growth
29	0.025	Prob: busi, celebr, small, communiti, #smallbiz, grow, #df19 FREX: small, busi, #smallbiz, celebr, trailblaz, owner, #shopsmal	Social Goal 11 Sustainable cities and communities
37	0.025	Prob: invest, market, explain, research, bank, listen, ahead FREX: bank, explain, invest, #podcast, ahead, europ, trend	Economic Goal 8 Decent work and economic growth
38	0.025	Highest Prob: patient, learn, research, cancer, treatment, new, studi FREX: medicin, cancer, treatment, patient, vaccin, clinic, trial	Social Goal 3 Good health and well-being
40	0.022	Prob: peopl, support, provid, learn, disabl, help, work FREX: disabl, peopl, provid, assist, affect, answer, languag	Social Goal 10 Reduced inequalities
24	0.022	Prob: ceo, watch, talk, video, founder, head, david FREX: founder, david, solomon, video, advic, interview, ceo	Economic Goal 8 Decent work and economic growth
28	0.022	Prob: health, care, help, awar, learn, rais, risk FREX: health, awar, care, heart, matern, #dyk, rais	Social Goal 3 Good health and well being
36	0.022	Prob: communiti, effort, respons, support, partner, impact, help FREX: effort, disast, natur, respons, communiti, suppli, chain	Social Goal 11 Sustainable cities and communities
11	0.020	Prob: innov, think, role, play, idea, big, challeng FREX: innov, idea, think, role, #innov, play, #pglifelab	Social Goal 9 Industry Innovation and infrastructure
23	0.020	Prob: #insideverizon, network, connect, #5 g, citi, break, speed FREX: #5 g, network, break, ultra, speed, wideband, #insideverizon	Social Goal 9 Industry Innovation and Infrastructure
32	0.019	Prob: join, career, meet, industri, learn, femal, entrepreneur FREX: femal, career, workshop, industri, meet, entrepreneur, pave	Social Goal 5 Gender Equality
4	0.019	Prob: offic, chief, brand, growth, marc, forc, economi FREX: chief, offic, marc, forc, brand, pritchard, economist	Economic Goal 8 Decent work and Economic Growth
27	0.017	Prob: improv, result, financi, valu, sale, strong, includ FREX: financi, result, sale, #earn, improv, strong, quarter	Economic Goal 8 Decent work and economic growth
14	0.016	Prob: visit, convers, step, inform, black, bias, campaign FREX: visit, bias, convers, inform, #talkaboutbia, racial, step	Social Goal 10 Reduced inequalities
39	0.016	Prob: opportun, advanc, initi, creat, workforc, cultur, skill FREX: initi, advanc, workforc, opportun, #saferchildbirthc, pathway, skill	Social Goal 11 Sustainable cities and communities
5	0.016	Prob: school, win, grant, high, award, vote, girl FREX: school, grant, vote, campus, win, preserv, compet	Social Goal 4 Quality education
25	0.016	Prob: digit, transform, nonprofit, close, gap, age, learn FREX: digit, transform, close, age, gap, rural, nonprofit	Social Goal 10 Reduced inequalities
8	0.015	Prob: sustain, read, director, ceo, releas, expand, execut FREX: sustain, jim, excel, director, expand, press, releas	Economic Goal 8 Decent work and economic growth
35	0.014	Prob: energi, speak, futur, ceo, carbon, mike, morn FREX: energi, carbon, mike, speak, shift, renew, emiss	Environment Goal 7 Affordable and clean energy

**Table 9:** Mixed and general CSR topics proportion, word composition metrics and CSR/SDG labels. The data are ordered by the Expected topic proportion. Source: Authors’ own elaboration

Topic	Exp. Prop	Top 7 stems
22	0.032	Prob: live, join, tune, tomorrow, week, question, ask FREX: tune, keynot, ask, tomorrow, #livefromverizon, question, live
30	0.026	Prob: like, keep, see, just, look, behind, #catrac FREX: #catrac, keep, like, run, race, scene, behind
13	0.020	Prob: show, experi, bring, good, light, team, love FREX: light, super, wonder, show, shine, london, peek
6	0.020	Prob: discuss, presid, #talksatg, realli, senior, leader, confer FREX: senior, realli, vice, john, general, former, #talksatg
45	0.017	Prob: tech, issu, forward, book, read, #toolsandweapon, look FREX: #toolsandweapon, issu, tech, smith, book, brad, forward
20	0.017	Prob: help, journey, organ, learn, resourc, can, other FREX: journey, hire, resourc, recruit, aim, organ, other
3	0.017	Prob: stori, drive, tell, passion, share, tree, stage FREX: stori, distract, tell, imagin, #everysecondmatt, tree, passion
34	0.012	Prob: report, full, hous, record, highlight, see, number FREX: report, record, incom, hous, net, number, reveal

**Table 10:** Non-CSR topics proportion, word composition metrics and CSR/SDG labels. The data are ordered by the Expected topic proportion. Source: Authors’ own elaboration

Topic	Exp. Prop	Top 7 stems
9	0.032	Prob: save, special, onlin, select, buy, space, today FREX: buy, special, save, onlin, space, select, outdoor
21	0.026	Prob: next, generat, design, cat, job, #letsdothework, oper FREX: generat, cat, job, oper, design, #letsdothework, next
43	0.024	Prob: custom, servic, new, platform, manag, cloud, consum FREX: custom, cloud, platform, servic, payment, trail, capabl
18	0.023	Prob: tip, know, season, follow, get, safeti, stay FREX: season, tip, safeti, fire, #thinksaf, stay, holiday
26	0.022	Prob: new, start, avail, core, time, hour, store FREX: core, start, processor, laptop, york, hour, avail
17	0.022	Prob: day, game, everi, favorit, kid, got, across FREX: game, favorit, gift, globe, kid, order, perfect
7	0.019	Prob: member, get, card, give, access, appli, now FREX: member, card, earli, tix, chanc, credit, thru
15	0.013	Prob: now, plan, pay, way, long, competit, applic FREX: competit, plan, transfer, choos, pay, applic, now
2	0.010	Prob: offer, readi, call, detail, get, click, limit FREX: offer, readi, click, limit, enrol, detail, call
1	0.008	Prob: appli, toward, term, point, receiv, long, newest FREX: point, toward, reward, arriv, #membershipreward, fli, newest
47	0.006	Prob: learn, today, take, share, new, strategist, one FREX: take, strategist, great, find, share, one, today

.

## Appendix 4: Covariates Effects

See Figures 7, 8, 9, 10, 11, 12, 13, 14.

## Footnotes

1 https://www.un.org/millenniumgoals/.

2 https://www.bloomberg.com/quote/INDU:IND.

3 https://www.msci.com/gics.

4 https://developer.twitter.com/en/products/twitter-api/academic-research.

5 See the section Published Applications on https://www.structuraltopicmodel.com/ for a full list of publications including the analysis of Twitter data.
